# Transforming our relationship with the social determinants of health: a scoping review of social justice interventions in Canadian Medical Schools

**DOI:** 10.15694/mep.2020.000191.1

**Published:** 2020-09-14

**Authors:** Nisha Kansal, Brittany Graham, Michael Kruse, Janice Lee, Anvita Kulkarni, Sureka Pavalagantharajah, Megan Chu, Jason Profetto, Albina Veltman

**Affiliations:** 1 McMaster University

**Keywords:** Social determinants of health, Clinical Skills, Social justice, experiential learning, transformative learning

## Abstract

This article was migrated. The article was marked as recommended.

**Background/Purpose**: Physicians are in a powerful position to improve the health status of communities through mitigating disparities rooted in social inequities. However, it is uncertain whether medical schools are preparing future physicians with the skills needed to care for diverse populations. The current scoping review aimed to describe how Canadian medical schools teach social justice, comparing pedagogical strategies.

**Methods**: A search was performed using OVID to identify published studies of implemented and evaluated social justice-based interventions within Canadian medical school curricula.

**Results**: Six studies were included. Common themes included increased content knowledge, greater understanding of SDoH, acknowledgement of power and privilege imbalances, identification of physicians’ roles as advocates, emphasis on the importance of interdisciplinary care, and increased capacity for self-reflection and personal growth. Experiential interventions were associated with greater personal transformation, but had limited accessibility.

**Conclusion**: Despite the widespread recognition of physicians’ roles as health advocates, there is a lack of consensus about an effective strategy for teaching social justice in medical education in Canada. While additional research focusing on the relative merits of didactic versus experiential learning is needed, these preliminary results suggest that experiential learning emphasizing self-reflection and personal growth may be optimal when approaching transformative learning.

## Introduction

It’s 1am in the emergency department and Mrs. S., a 58-year-old lifetime smoker with COPD who is well-known to the ED staff is brought by ambulance with difficulty breathing. Paramedics tell you that they pick her up from her apartment almost weekly on the “rough side of town” where she lives alone, and you wonder why this keeps happening.

As you try to get a more comprehensive history, you find out that Mrs. S is a First Nations woman who struggles to live on her disability pension after suffering a fall 5 years ago that left her unable to even volunteer at her local Indigenous youth centre. She tells you of the loss of her husband, a residential school survivor, who overdosed 6 months ago after suffering for years from a polysubstance addiction. She admits to a loneliness and isolation that you can’t imagine, and you wonder not only how all of these struggles are impacting her life, but also what you as a medical student can possibly do to help.

In recent years, the medical profession has come to recognize the importance of social determinants of health (SDoH), defined as “the conditions in which people are born, grow, live, work, and age..these circumstances are shaped by the distribution of money, power, and resources at global, national, and local levels” ( World Health Organization, 2019). In Canada, 50% of a person’s disease burden is related to their SDoH, including income, early childhood development, race, gender, sexual orientation, housing, education, and a myriad of other factors ( Tyler, 2019). However, the healthcare system often lacks mechanisms to address these factors. Insufficient education around SDoH at the medical school level likely contributes to these gaps; students are rarely taught about the history that has produced current conditions, issues within the healthcare system that perpetuate inequities, and particularly what we can tangibly do to produce change at the individual and community levels ( Sharma, Pinto and Kumagai, 2018). Social injustice, created and mediated by the larger forces of racism, colonialism, classism, and capitalism, is ultimately responsible for producing differential health outcomes through downstream social divisions based on SDoH. These forces manifest in people, institutions, and social systems, born out of cultural, political, and economic pressures. In working to improve the health of individuals and populations, it becomes the responsibility of future healthcare providers to identify the root causes of these pressures, and ultimately by doing so, efforts can be made to dismantle them. An improvement and emphasis in teaching around these forces would likely mitigate against their reinforcement through the healthcare system over time.

In recognizing the need to provide equitable care to all patients, particularly those historically and currently marginalized by our systems, this scoping review was conducted to identify and describe social justice-based teaching interventions integrated into the curricula of Canadian medical schools. As explained in the framework proposed by Arksey and O’Malley (2005), a scoping review is undertaken to understand the extent and range of knowledge in a particular field of research, disseminate those findings quickly, and identify any gaps in the literature. This may be done to determine the value of undertaking a larger and more robust systematic review. Our goal is to inform the development of future interventions for medical school curricula.

## Methods

### Search strategy

**Figure 1.**
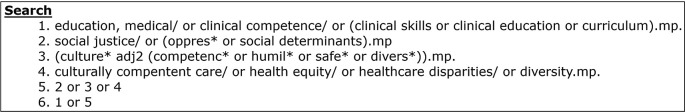
Search Criteria

Using the Ovid database for Medline from 1946-present, an electronic search was performed using Keywords including “education”, “medical”, “clinical competence, “social justice”, and “health equity”. See Figure 1 for complete search details.

### Study selection

Study selection was conducted in three rounds of screening: title, abstract, and full text. Similar inclusion and exclusion criteria were applied to each stage. Included articles discussed formal medical curricula changes directed toward working with diverse patient populations. Originally, study populations included were healthcare professional students of any profession (e.g. medical students, residents, nursing students, physiotherapy students, and other allied health professionals). After the search was conducted, the population was narrowed to only include medical students and residents in Canada, to increase the applicability of the findings to medical schools in the national context. Studies that measured outcomes of knowledge and skills in working with diverse populations as well as student comfort, confidence, and experience were included.

Exclusion criteria included interventions focused on affirmative action, broad pedagogical perspectives without concrete changes to curricula, articles in languages other than English, and articles whose abstracts were not available through the McMaster University library.

The review process was conducted such that each article was reviewed by two reviewers independently at each stage, who then met to discuss discrepancies. In the abstract and full-text review stage, a neutral third party from the group was involved in the final decision to resolve conflicts.

### Data extraction

Data was extracted regarding demographics of the article, population, objectives, intervention type, methods and results ( Table 1).

### Summary methods

In this qualitative scoping review, key themes were identified in each article included. Common themes and strategies were then grouped together for the thematic analysis.

**Table 1.** Data extraction tool

Section	Criteria
Study information	Title
	Abstract
	Location
Population	Population (i.e. medical students, pre-clerkship vs. clerkship, residents)
Objectives	Objectives were paraphrased
Intervention	Intervention time course
	Intervention focus (what social justice concept was the intervention targeting?)
	Learning style (e.g. lectures, small group, experiential, observership, etc.)
	Mandatory vs. elective
	Broad overall description of the intervention
Methodology of the study	Study design (e.g. pre/post survey)
	Follow up (Yes/No/length of time)
	Measures
	Broad overall description of methods
Results	Overall description of results
Conclusion	Conclusion of the study

## Results

**Figure 2.**
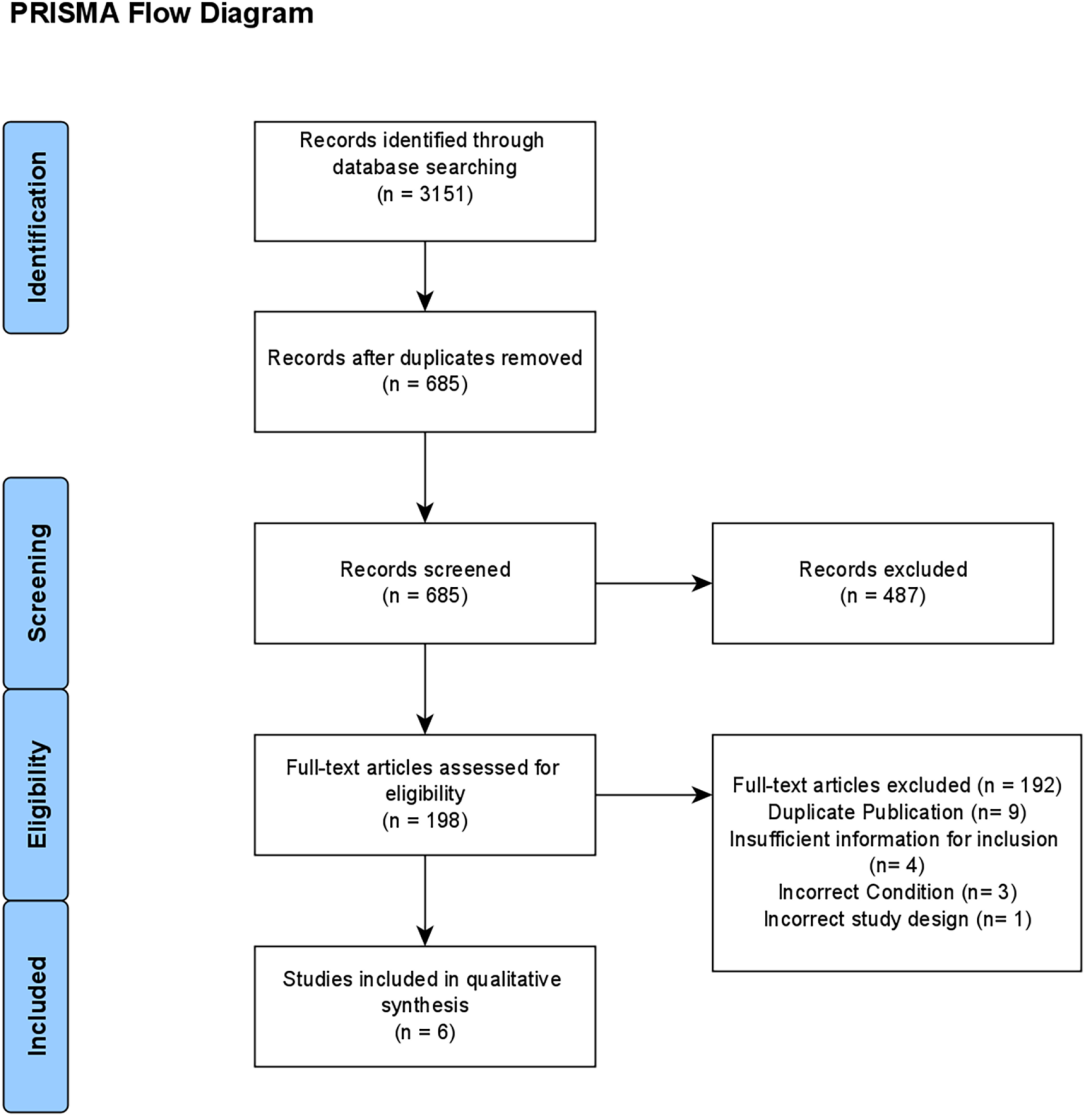
PRISMA Flow Diagram

A total of 3151 articles were identified through the initial database search ( Figure 1). After duplicates were removed, 685 articles remained, of which 198 articles were included for full-text screening. After the initial full-text screening, 135 articles remained. At this point, the inclusion/exclusion criteria were narrowed from any study involving medical, dental, or nursing students on a global scale to those involving only medical students in Canada. A total of six articles remained after this change, which is summarized in the PRISMA flow diagram ( Figure 2).

Six common themes were identified amongst the papers, as outlined below. Table 2 summarizes the interventions, evaluation methodology, and the results of each paper.

Of the six studies analyzed, two focused on service-learning, two included a mix of experiential and didactic learning, and two included purely didactic teaching methods. The Making the Links course at the University of Saskatchewan (UofS) included three different immersive experiences ( Meili, Fuller and Lydiate, 2011). Students spent six weeks in remote communities in the north of the province, volunteered in an urban-health clinic in Saskatchewan, and spent an additional six weeks in Mozambique. The international service-learning experience at the University of British Columbia (UBC) also included a clinical experience abroad, which comprised of eight weeks in Uganda ( Dharamsi *et al.*, 2010). Due to the resource-intensive nature of these programs, spots were open to a limited number of students. Fourteen students participated in the Making the Links project ( Meili, Fuller and Lydiate, 2011), while only three UBC students travelled to Uganda ( Dharamsi *et al.*, 2010).

The Social Pediatrics Elective ( van den Heuvel *et al.*, 2014) and the Pre-clerkship HIV Elective ( Chew *et al.*, 2012), both at the University of Toronto (UofT), included clinical exposure to the topics, complemented with assigned readings, group discussions, and lectures. Similarly, these opportunities were available to a limited number of students.

By contrast, purely didactic courses, such as the Physicians, Patients and Society (PPS) course at Dalhousie University ( Beagan, 2003), and the Interdisciplinary Population Health Project (IPHP) at UofS ( Racine, Proctor and Jewell, 2012) were open to a much larger number of students. The PPS course included lectures and small group discussion on a variety of topics related to the social context of medicine, while the IPHP course focused on learning to work in interdisciplinary teams, engaging in community development to address health challenges, and locating SDoH within their broader sociopolitical context.

### Theme 1: Acquired content knowledge and applicability of learning

Three of the six papers included in this review discussed the theme of acquiring knowledge through didactic sessions and the skill of applying this knowledge in a clinical setting. In the social pediatrics elective at UofT that was discussed by Van den Heuvel *et al*. (2014), the authors found that students participating in this elective gained specific knowledge about the topics that were circulated in order to guide their reflection papers. Example topics included prevention, experience-based brain development, and suboptimal life trajectory. Similarly, Racine *et al.* (2012) found that through the implementation of the IPHP at the UofS students increased their knowledge of population health and community development concepts.

Chew *et al*. (2012) noted that the pre-clerkship HIV elective created at UofT increased participants’ confidence in serving populations affected by HIV; it was able to do so through teaching students more about the psychological and medical aspects of HIV, filling a knowledge gap within the curriculum. Additionally, the students appreciated the breadth and diversity of topics covered by the elective and found the interactive components more valuable than didactic teaching.

Dharamsi *et al.* (2010) investigated students’ experiences on an international service-learning experience through UBC, and found that through experiential learning, students found it easier to understand concepts that seemed abstract in a classroom setting.

### Theme 2: Greater recognition and understanding of SDoH

Four of the six papers found that students developed a greater recognition and understanding of SDoH through their elective experiences. The UofT social pediatrics elective implemented by Van den Heuvel *et al.* (2014) found that SDoH was the most commonly described theme in the reflective papers written by participants. In particular, they began to understand the interplay between poverty and health and were exposed to diverse patient populations. Similarly, students who participated in service-learning experiences through UBC ( Dharamsi *et al.*, 2010) and the Making The Links program through UofS ( Meili, Fuller and Lydiate, 2011), found that experiential learning helped them build a greater appreciation for the SdoH and their impact.

Racine *et al.* (2012) found that through the implementation of the IPHP at UofS, students generally increased their knowledge of SDoH. Students commonly felt that their previous understanding of SDoH was quite abstract and lacking until experiencing these various programs and electives.

### Theme 3: Understanding and acknowledgement of privilege and power imbalances

Four papers identified the theme of privilege and power imbalances as relevant topics to medical education. The social pediatrics elective at UofT ( van den Heuvel *et al.*, 2014), international service-learning experiences at UBC ( Dharamsi *et al.*, 2010) and the Making The Links program at UofS ( Meili, Fuller and Lydiate, 2011) taught students about the dynamics of patient-physician relationships within the context of power and privilege. Van den Heuvel *et al*. (2014) found that participants acknowledged their own privileged backgrounds in their reflections and recognized the difficulties that children in impoverished settings face when they begin their lives with barriers rooted in poverty. The UBC students from the Dharamsi *et al.* (2010) paper challenged the stereotypes of and recognized the strength, resilience and determination that individuals in impoverished communities have. Similarly, participants in the UofS Making The Links Program ( Meili, Fuller and Lydiate, 2011) reflected on the patient stereotypes they held while interacting with individuals in the communities. After completing the course, they identified the stigma that certain populations face, such as Indigenous populations, and realized the importance of their relationships with patients and communities. Furthermore, students in both programs recognized the power imbalance that exists in relationships between different health professionals, as well as in the patient-physician relationship.

By contrast, implementation of the PPS course at Dalhousie University did not significantly change the percentage of students who believed that social factors such as class, race, culture, gender, and sexual orientation affected their experiences in medical school or their interactions with patients ( Beagan, 2003). Both before and after implementation of the course, most students did not feel that social factors were significant considerations. Interestingly, both before and after implementation of the PPS course, students who belonged to minority or vulnerable social groups were more likely to think that social and cultural factors affect medical practice, as compared to students from more socially privileged groups. For example, students who identified as poor were more likely to identify class as influencing their experience as medical students than students who did not. Membership in a socially marginalized group, and not participation in the PPS course, was a predictor of the students’ acknowledgement of privilege and power imbalances.

### Theme 4: Identification of the role of healthcare professional as an advocate

Two of the six papers identified that students recognized the role of healthcare professionals as advocates. Van den Heuvel *et al*. (2014) found that the UofT social pediatrics elective helped students recognize that part of their role is to work with parents and advocate for their child as a team. Similarly, UBC students in the Dharamsi *et al*. (2010) study were asked to study the CanMEDS document and identify how their role as an advocate can be learned. Previously, they described this role as “some of the more elusive themes in medical education” but found that their role as an advocate “is indeed fostered and redefined by experience”. They learned about social responsibility and how it involves more than simply donating to charity: it includes creating relationships and helping to amplify the voices of the vulnerable.

### Theme 5: Importance of interdisciplinary care

Four of the six papers found that students recognized the importance of interdisciplinary care through their respective electives and programs. Van den Heuvel *et al*. (2014) found that although there were no specific learning objectives or pre-circulated readings about multidisciplinary care, students involved in the UofT social pediatrics elective learned about the importance of teams working together and were more aware of healthcare as a system.

Students from the UofS Making The Links project ( Meili, Fuller and Lydiate, 2011) reflected on and understood interdisciplinary care in the context of the hierarchy of medicine, particularly in underserved areas. They realized that in low resource areas, interdisciplinary care is especially important and community development is a way to meet community needs in culturally appropriate ways.

Chew *et al*. (2012) found similar results in that students in the UofT pre-clerkship HIV elective developed an increased awareness of community and interdisciplinary management of HIV. They learned about interprofessional cooperation in healthcare, including healthcare professionals such as doctors and nurses working with community agencies, naturopathic doctors, and lawyers.

Interestingly, Racine *et al*. (2012) found that though their medical students appreciated the opportunity to work with students from other disciplines at UofS, they did not demonstrate increased readiness to participate in interprofessional healthcare teams. Additionally, they were less likely to agree that it is important to study population health with other health science students compared to kinesiology and physiotherapy students.

### Theme 6: Self-reflection and personal growth

Three papers recognized the importance of self-reflection and personal growth that students experienced. Eight of the 18 students in the UofT social pediatrics elective ( van den Heuvel *et al.*, 2014) experienced transformative learning, defined by the study authors as “the process of using a prior interpretation to construct a new or revised interpretation of the meaning of one’s experience in order to guide future action and decision-making”. Self-reflection was identified as a key element of this process. A similar theme was identified by Dharamsi *et al*. (2010) and Meili *et al*. (2011), where critical reflection was identified as enhancing learning and overall personal growth.

**Table 2.** Summary of Papers from Literature Search (n=6)

Study identifier	Intervention	Course topics	Evaluation Methodology	Relevant Findings
Baegan (2003) Dalhousie University “Physicians, Patients & Society (PPS” n=133	Weekly small group tutorials with occasional guest lectures	-Sociology of medicine -Sexual medicine -Complementary and alternative medicine -Population health and health promotion -Domestic violence -Mental health, addictions -Epidemiology, -Health policy -Ethics and law in healthcare	Questionnaire and interviews with third-year medical students prior to implementation of PPS course and three years after implementation	Most students reported that social group membership did not significantly impact their experiences as medical students, or the physician-patient relationship regardless of whether they completed the PPS course
Chew, Jaworsky, Thorne, Ho, Andany, Morin, Hoffman, Henshaw, Rourke, Fisher & Rachlis (2012) University of Toronto “Pre-clerkship HIV elective” n=18	-Student-designed and led pre-clerkship elective -Lectures, small group sessions, reading assignments, clinical observerships community agency placements, and HIV counselling and testing workshop -Clinical observerships -Community agency placement -HIV counselling and testing workshop	-HIV prevention -Mental health, stigma and discrimination of those living with HIV -Special populations affected by HIV -International perspective on HIV/AIDS	Pre- and post-questionnaires to evaluate self-perception of HIV knowledge Qualitative analysis of participants’ reflections	Students reported increased confidence in serving populations affected by HIV Student-led initiative effectively enhanced the medical curricula.
Meili, Fuller & Lydiate (2011) University of Saskatchewan “Making the Links” n=14	-20-hour orientation to health issues of underserved -Northern community experience for 6 weeks -Volunteer experience at a student-run clinic in an underserved urban area -International experience in Mozambique for 6 weeks -Reflection and evaluation of experience	-Aboriginal health -Urban health -Health of remote/rural communities -Global health -Languagetraining	Questionnaires following northern and international experiences -Reflection Piece	Students reflected on the importance of relationships with patients and communities to deliver culturally appropriate care. They also reflected on the limits of a disease-centred approach to medicine and the importance of SDoH
Dharamsi, Richards, Louie, Murray, Berland **,** Whitfield, and Scott (2010) University of British Columbia “International Service-Learning Experience” n=3	-8-week trip to Uganda to engage in a collaborative experience -Critical reflection through journaling	-Global health -CanMEDS health advocate role	Thematic analysis of post-exposure journals and essays	Experiential learning and critical reflection play a key role in developing health advocacy skills Students gained a greater understanding of socially responsible community engagement and what it means to be marginalized and vulnerable
Racine, Proctor, and Jewell (2012) University of Saskatchewan “Interdisciplinary population health project” n=158	-Small interdisciplinary groups including medical students, nursing students, kinesiology students and physiotherapy students -Weekly 2-hour facilitated learning activities including team building, community work, client interviews with volunteer guests, and panel discussions	-Develop Skills and attitude for interdisciplinary teamwork -Apply existing data and community health resources to solve population health issues. -Understand the social, political and economic context of health.	Pre- and post- surveys to evaluate changes in perception towards underserved populations, readiness to work in interprofessional teams, and subjective experiences or feedback	There was a significant difference of attitudes between students from different programs regarding the importance of interprofessional learning Student feedback indicated a necessity to increase the number of hours spent on marginalized communitiesCollaboration with community partners is necessary to prevent further marginalization of these communities
van den Heuvel, Au, Levin, Bernstein, Ford-Jones, Martimianakis (2014) University of Toronto, “Social Pediatrics Elective” n=15	-3-week clinical elective with assigned readings, group tutorials and self-reflection	-Failure to thrive -Infant attachment and its role in experience-based brain development -Child protection and maltreatment -Substance use and its effect on pediatric populations -Multicultural communities	Narrative analysis of self-reflection and comparison with elective objectives	A structured clinical experience and narrative reflection can change the assumptions and understanding of SDoH in 3rd and 4th year medical students

## Discussion

This scoping review of educational approaches implemented to improve social justice-based teaching in Canadian medical schools suggests that this content has been delivered through didactic, discussion-based, and service-learning programs with varying success. Self-reported outcomes on student learning tended to be favorable across all studies regardless of the teaching method. However, most qualitative evaluations of service-learning interventions found that students’ worldviews on issues of social justice changed as a result of their experiences ( Dharamsi *et al.*, 2010; Meili, Fuller and Lydiate, 2011; van den Heuvel *et al.*, 2014) whereas studies on didactic interventions either did not make this claim ( Racine, Proctor and Jewell, 2012) or found that the intervention had no effect on students’ worldviews ( Beagan, 2003).

Previous authors have argued that changing students’ worldviews is an essential part of teaching social justice and social accountability. Proponents of this “transformative learning” believe that fluency in the humanistic values of medicine requires “stepping back to understand one’s own assumptions, biases, and values and a shifting of one’s gaze from self to others and conditions of injustice in the world” ( Kumagai and Lypson, 2009). Cavanagh, Vanstone, and Ritz (2019) discuss the potential for transforming medical education through Paulo Freire’s approach of Critical Pedagogy, best-known from his *Pedagogy of the Oppressed* ( Freire, 1970). Critical pedagogy views education as a “practice of freedom” to develop and awaken “critical consciousness” amongst learners through self-directed learning that allows for the exploration of conditions leading to social inequities. Freire’s work proposed that individuals with critical consciousness would be encouraged to subsequently bring about change in their world through political action and social justice work ( Freire, 1970). Despite a consensus about the importance of transformative learning in medical education, relatively little is known about the best way to implement this pedagogy. However, most authors agree that students must be active participants in their learning in order to experience a fundamental shift in their worldview ( Cavanagh, Vanstone and Ritz, 2019).

Service-learning is a way of teaching wherein students work directly with community members and use reflection as a tool to discover organic connections between their experiences and the formal educational objectives of the curriculum. A systematic-review of service-learning in medical education found that this approach has been used by educators for a variety of reasons, including improving interpersonal skills, developing critical thinking, in addition to increasing students understanding of health disparities and how they can be addressed ( Stewart and Wubbena, 2015).

The results of the current review suggest that the reflective component of service-learning may be particularly well-suited to encourage transformative learning. The studies by Dharamsi *et al.* (2010), van den Heuvel *et al.* (2014), and Meili (2011) all identified that students’ self-reflections named some element of transformation as a result of their experiences. The two-year Making the Links project at UofS represents the most immersive of the service-learning interventions included in this review. Qualitative analysis of student reflections revealed a shift in how students understood the importance of culturally competent medical care and the “limitations of a disease-centered approach” ( Meili, Fuller and Lydiate, 2011). Furthermore, students described a shift in their thinking about the role of social position in the physician-patient relationship.

By contrast, didactic interventions included in the current study did not routinely incorporate reflection as part of the curriculum. An interesting finding from the study by Beagan (2003) about the Physicians, Patients & Society course at Dalhousie University was that students’ perception of the significance of social group membership on the physician-patient relationship was not significantly altered after implementation of the course. This suggests that the course had little effect on the students’ worldviews. The finding that membership in a socially disadvantaged group increased the likelihood that students would recognize that particular social factor as affecting their lives as students, the lives of their patients, and patient-physician interaction could suggest that lived experience is an important factor influencing students’ perception of social justice: perhaps, more important than medical school curriculum. Although students who participate in experiential learning will never have the knowledge that those with lived experience have, this type of learning may be the closest approximation, and thus, the intervention most likely to lead to meaningful, transformative change in their worldviews.

## Limitations & areas for future research

While the current review provides support for the role of active participation and critical reflection in developing medical students’ understanding of social justice in medicine, more robust evidence about the role of service-learning in teaching social justice is needed. There are several limitations of these studies. Sample size and recruitment are the most limiting aspects. However, lack of long-term follow-up and a reliance on self-assessments for evaluation, as well as a lack of control groups, prevent generalization from the data.

In the four studies of experiential learning, small sample sizes were the norm, with 14-21 students in three studies and only three students in Dharamsi *et al.* (2010). These small sample sizes are understandable when we consider the limited number of positions available to learners to work in the community and the funding required to support a student in an international or rural elective. A 2015 review of other service-learning programs found funding to be the most significant barrier to participation ( Stewart and Wubbena, 2015). However, the fact that participants in these studies either applied or were self-selected also introduces the possibility of positive selection bias skewing the results. These students may have had an awareness of the role of power and privilege in patient-physician relationships prior to applying. The interventions may not have created transformative learning as the authors claim, but rather reinforced pre-existing attitudes among the selected sub-population of students. By contrast, in the studies implementing didactic interventions by Beagan (2003) and Racine *et al.* (2012), participation of all students was mandatory, resulting in greater numbers of participants (61-158). The fact that these studies found either minimal or no change in students’ orientation to principles of social justice may not simply be due to the inadequacy of didactic pedagogical methods. Rather, it may be a consequence of the diverse participant group, which likely included those who were disinterested and disengaged from the subject matter. As a result of these limitations, the studies do not conclusively identify the overall best pedagogical method to optimize engagement and learning of the entire student body. Further research in this area is vital.

None of the studies examined in this review used contemporary control groups, so it cannot be discerned whether there were other cultural or didactic factors that would have led to a change between pre- and post-testing. Beagan (2003) did conduct a survey three years prior to the intervention in a similar cohort and used this as a comparison to the intervention group. It should be noted that in the four studies that had elective participation, the students were self-selected to participate. These students were therefore primed to be open to change and it could be argued that they had a high chance of already holding values that were close to or consistent with the program objectives. In two of the studies by Chew *et al*. (2012) and Dharamsi *et al.* (2010), there was an interview and selection process used that could add further bias into the selection process, with the latter only choosing three students in which two had prior experience in low-resource settings. Selection may be important to ensure the safety of communities in which students will be entering, but also makes it a challenge when trying to generalize these findings to the average medical student. It is important to note that voluntary participation has been identified as an important component of a constructive experience ( Muller *et al.*, 2010), while elective and selective programs often had students who were predisposed to be altruistic and service-oriented ( Stewart and Wubbena, 2015).

Evaluation varied among the studies, but they all relied on a subjective self-assessment, which is a weaker marker of change in comparison to external evaluation. Three of the studies only focused on post-interventional assessment ( Dharamsi *et al.*, 2010; Meili, Fuller and Lydiate, 2011; van den Heuvel *et al.*, 2014), which limits the ability to assess for change. Additionally, there is a performative aspect of the self-evaluation process whereby the student may wish to please the evaluators and support the program, especially if they valued their experience. Moreover, there was no long-term follow-up to determine if the changes were robust and maintained throughout residency and beyond. More objective and standardized measures need to be developed in order to identify an intervention that results in real change in social justice domains, and future studies should include longitudinal testing to determine whether the change in individual attitudes result in changes to medical practice ( Meah, Smith and Thomas, 2009; Stewart and Wubbena, 2015; To and Sharma, 2015).

Finally, none of the studies in this review assessed the experiences of the patients or community partners. As a result, it is important to consider that there may not have been utility of these programs for each community, and perhaps even unidentified harm done by these programs to the patients and community partners. Many of the populations that are served in these service programs are already vulnerable and may rely more than the general population on student-run or -staffed clinics for their healthcare. Lahey (2012) identified the risk of substandard care by trainees to these populations, and future studies should incorporate a longitudinal component to outcomes to identify any possible adverse effects and discuss further any ethical implications. Additionally, this data would give a more objective measure of the students’ change in values or behaviours that are supportive to the community they are serving.

The original search in 2017 found 3151 papers. It is important to note that if the search was run in November of 2019, we would find a total of 3749 results. This leaves an additional 598 papers we did not evaluate. Given the findings of 6 relevant papers in the original search. It is unlikely that more than 1 or 2 additional papers would be found today. Therefore, we can be confident that our current scoping review captures the bulk of relevant papers.

Given the concerns discussed above, it is likely difficult for a student not previously motivated to broaden their experience and understanding of social justice domains to reflect upon their privilege in a manner that is “transformative”. These studies do not suggest how the transformation in thinking and values occurred, or even if it occurred at all. Beagan (2003) and Racine *et al*. (2012) studies showed that didactic learning is not enough to change values or increase understanding of social justice in a critical way, while other studies showed that students who are motivated to work in these communities are ultimately the ones having transformative experiences. Furthermore, none of the studies revealed what happens when students who may be neutral toward social justice domains are given experiences that challenge their ideas about social justice. This is the group with the biggest potential for change.

## Conclusion

Given the significant health disparities experienced by numerous groups marginalized by our society and social structures, current and future healthcare providers have an imperative to practice in a manner that would ultimately create health equity. Physicians are in a position of social power and as such, will have significant influence to stimulate change. Medical education should thus be regarded as a locus of change to educate future healthcare providers on how they may improve the health of their patients in a holistic manner, treating both biological and social roots of illness. The studies reviewed highlighted the key concepts of experiential learning, reflective practice, and “transformative learning” as processes that could be engaged in during medical school to likely contribute to more socially-just medical practice.

By expanding students’ worldviews of the causes of illness in the lives of their patients, it is possible that one could tangibly contribute to the provision of more equitable healthcare. Challenges persist in the implementation of medical curricula that would allow for the execution of this process; however, the literature demonstrates this to be a key and worthwhile pursuit. Medical students and future healthcare providers have a duty to act in a socially accountable manner to all communities relying on them for better health; it is our responsibility to equitably provide the care that everyone in Canada needs.

## Take Home Messages

• Medical education is crucial in educating future healthcare providers on how to care for patients holistically.
• Current social justice-based teaching is delivered through didactic, discussion-based, and service-learning programs with varying success.
• Experiential learning, reflective practice, and “transformative learning” in an undergraduate medical school curricula are processes that could contribute to more socially-just medical practice.

## Notes On Contributors

Nisha Kansal: Dr. Kansal is a Family Medicine Resident Physician at McMaster University with an interest in improving the health of marginalized communities. She aims to use medicine as a tool for social justice through clinical work, research, and systems-level activism, with a particular focus on migration and health.

Brittany Graham: Dr. Graham is a graduating medical student at McMaster University and is a resident in Family Medicine/Public Health and Preventive Medicine. Prior to medical school, she completed graduate studies in Global Health Science and Social Anthropology at the University of Oxford.

Michael Kruse: Dr. Kruse is a graduating medical student at McMaster Medicine, and is a Family Medicine Resident at Queen’s University. His research interestes include LGBTQI+ care in the pre-hospital and emergency environents. ORCID: https://orcid.org/0000-0001-9519-9873.

Janice Lee: Dr. Lee is currently a family medicine resident at University of Toronto. She is passionate about the people in front of her and the communities that surround them.

Anvita Kulkarni: Dr. Anvita is a family medicine resident at Queen’s University (Kingston site). She completed her medical training at McMaster University. Prior to that, she completed an MPH at the Harvard Chan School of Public Health.

Sureka Pavalagantharajah: Ms Pavalagantharahah has a BHSc (honours) from McMaster University and is an MD candidate at the Michael G. DeGroote School of Medicine who is passionate about medical education, advocacy and health equity.Megan Chu: Ms Chu is a medical student at the Michael G DeGroote School of Medicine, who completed her Bachelors of Health Sciences degree with honors at McMaster University. She has an academic interest in trauma, social determinants of health, and medical education.

Jason Profetto, BKin, MD, CCFP: Dr. Profetto is a McMaster Medicine graduate and alumni who also completed his Family Medicine Residency through McMaster. He continues to be actively involved in the Undergraduate Medical Program at McMaster as both the Chair of Clinical Skills and a teacher for all levels of medical students.

Albina Veltman MD, FRCPC, BSc (Hons): Dr. Veltman is a Associate Professor, Department of Psychiatry & Behavioural Neurosciences and former Diversity & Engagement Chair, Undergraduate MD Program, McMaster University. Dr. Veltman’s clinical work focuses on traditionally marginalized populations, including people with severe and persistent mental illness, people with developmental disabilities, and people who identify as LGBTQ.
