# Supplementary material for: Transforming our relationship with the social determinants of health: a scoping review of social justice interventions in Canadian Medical Schools
Source: MedEdPublish (2016). 2020 Sep 14;9:191. [Version 1] doi: 10.15694/mep.2020.000191.1 (PMC10699394; doi:10.15694/mep.2020.000191.1)
Supplement: Peer review history

# Reviewer report: Reviewer response for version 1

**DOI:** 10.21956/mep.20083.r30918

**Published:** 2020-09-17

**Authors:** Keith Wilson

**Affiliations:** 1 Dalhousie University

This review has been migrated. The reviewer awarded 5 stars out of 5 The present study examined the existing literature to determine how social justice interventions were being explored in Canadian medical schools. It is important for the reader to recognize that this is a scoping review of existing published literature and is not (necessarily) a snapshot of current initiatives. The authors highlight the strengths and limitations of the studies currently published and make a call for further research in this area. For the purposes of this study, they focused on articles pertaining to education of medical students and residents. Their approach to analyzing the articles is well laid out although the reader may have wanted to know more about what qualitative approach was used (and perhaps some more detail about the iterations). The themes that emerged have face validity and certainly push educators into considering them in curricula development. In particular, those involved in health professions education can relate to themes like interdisciplinary care, advocacy, self-reflection and privilege.Despite the low number of articles identified in the published literature on the topic, the authors delve deeply into them and provide an incredibly rich analysis of the articles, subsequent themes and implications.This is a well written article with very insightful and nuanced observations of what is and what should be done in relation to ongoing education in this area. The authors should be commended for this excellent article. This should be read by anyone involved in curricula development using a social justice lens.

Reviewer Expertise:

NA

No decision status is available

# Reviewer report: Reviewer response for version 1

**DOI:** 10.21956/mep.20083.r30917

**Published:** 2020-09-15

**Authors:** Trevor Gibbs

**Affiliations:** 1 AMEE

This review has been migrated. The reviewer awarded 5 stars out of 5 I really enjoyed reading this paper, covering a very important element within the curriculum and one we need to pay constant attention to, now more than ever. I found the methodology to be appropriate at this level and as expected the number of papers dealing with this specific problem were scarce. I would recommend this paper to all those involved in curriculum planning

Reviewer Expertise:

NA

No decision status is available
